# Endometrial abnormalities on transvaginal ultrasonography and histopathology in women after quinacrine sterilization

**DOI:** 10.12669/pjms.304.5091

**Published:** 2014

**Authors:** Saira Afzal, Mulazim Hussain Bukhari

**Affiliations:** 1 Dr Saira Afzal, MBBS, MCPS, M.Phil, FCPS, Chairperson and Head, Department of Community Medicine, King Edward Medical University, Lahore, Pakistan.; 2 Prof. Mulazim Hussain Bukhari, MBBS, MCPS, M.Phil, FCPS, PhD, Deputy Dean, Pathology Department, Medical School.American University of Barbados, School of Medicine 18 Wildey Estate, Main Wildey Road, St Michael, Barbados, USA.

**Keywords:** Quinacrine sterilization, Endometrial pattern, Sterilization failure

## Abstract

***Objective: ***To describe endometrial abnormalities on transvaginal ultrasonography and histopathology in women after quinacrine sterilization.

***Methods: ***It was an analytical cross sectional study conducted during February 2012 to April 2013. The sample size calculated at 95% confidence level was 540. Sampling technique used was simple random sampling. The medical history, examination, transvaginal ultrasonography and biopsy of suspected lesion was performed in quinacrine sterilized women.

***Results: ***The calculation of statistics showed the mean age at quinacrine sterilization was 38.5 years, standard deviation 6.517, and standard error 0.461. The endometrium was regular and smooth with homogenous images in 86% (n= 466), irregular endometrium with heterogeneous images on transvaginal ultrasound in 9.4% (n =51) and endometrial growth with high level echoes in 4.2% women (n= 23). The histological findings included hyperplasia and well differentiated adenocarcinoma in two patients respectively.

***Conclusion: ***The irregular endometrium, adhesions, and growths were found after quinacrine sterilization. The risk of endometrial growth was more after 10 years duration of quinacrine sterilization.

## INTRODUCTION

Quinacrine is a very old drug that is used for many years by humans for many medical conditions including treatment of malaria, arthritis, giardiasis^1^, Systemic lupus eryhtematosus^2^, leishmaniasis, pleurodhesis in lungs^3^ and female sterilization by blocking the fallopian tubes.^4^ The quinacrine sterilization was found cheap, easily performed and acceptable to the females in comparison to surgical sterilization method.^5^ The quinacrine sterilization has been performed in more than 175,000 women all over the world including Singapore, Thailand, Vietnam, Brazil, United States of America, China, Indonesia, India and Pakistan. More than 11,000 women underwent quinacrine sterilization in Faisalabad Pakistan.^6^

Quinacrine insertion was performed by inserting quinacrine pellets in the uterus by intra uterine device inserter under aseptic conditions and all pellets were released in a line near fundus of the uterus during proliferative stage. Same procedure was repeated after one month duration for double insertion. 90% women opted for single insertion of quinacrine. Only 10% had double insertion of quinacrine in Faisalabad.^6^

The official statistical maternal mortality in Pakistan was 286 deaths per 100,000 live registered births.^7^ The maternal morbidity and mortality was improved after quinacrine sterilization in developing countries.^8^ The data collected in follow-up of the participants identified a cluster of cancer cases among women who had received quinacrine but it was statistically insignificant.^9^^,^^10^

World Health Organization (WHO) had suggested that there should be continued surveillance of women who have received quinacrine sterilization in the past for risk of health complications, such as endometrial adhesion-related morbidity, or adverse maternal and fetal outcomes.^11^

Nevertheless, many peer reviewed research studies had suggested that quinacrine sterilization was relatively safer than surgical sterilization but scanty literature was available about transvaginal ultrasonography and endometrial histopathology after quinacrine sterilization. Our aim was to identify endometrial abnormalities like adhesions and growths after intrauterine insertion of quinacrine for female sterilization on transvaginal ultrasonography and histopathology.

## METHODS

This population based cross sectional study was conducted in Faisalabad, Pakistan. The ethical issues were considered, informed consent was obtained and all information was kept confidential. The ethical permission was obtained from Advanced study and Research Board King Edward Medical University. 

To find the optimal sample from the study population, the sample size calculator Epi info2000 software was used. The study population of quinacrine sterilization was 11000.^6^ The expected frequency of endometrial adhesions in quinacrine sterilization was 6.4%.^12^ The worst acceptable result was 8.45% used for calculations. Sample was calculated at 95% confidence level and 80% power of the test. According to this formula, the sample used in the study was 540 subjects who were selected randomly from the study population. 

**The women with history of hormonal treatment, pelvic inflammatory diseases, polycystic ovary syndrome were excluded to control bias. **Endometrial thickness was measured from echogenic border to echogenic border across the endometrial cavity in a sagittal midline plane by transvaginal ultrasound. The double layer of endometrium less than 5mm thick and no focal thickening was defined as thinning of endometrium. Homogenous, regular, smooth endometrium measuring 5-7mm was considered as normal endometrium. If endometrial thickness was 8mm or greater it was described as thickened endometrium. Endometrial adhesions were defined as thick fibrotic bands that had distorted the uterine cavity. On transvaginal scan they were reported as heterogeneous, hyperechoic, punctated shadows and irregularities. Hyperplasia was visualized as diffuse or focal hyperechoic endometrial thickening or asymmetrical heterogenous images and irregularity. Endometrial abnormalities and irregular bleeding per vagina were advised biopsy under ultrasound guidance by a gynecologist. The biopsy was obtained under aseptic conditions. The histopathology of the specimen was done in histopathology section of Pathology Department in King Edward Medical University Lahore by three histopathologists. Each histopathologist gave independent report to control bias.

Data was analyzed using Statistical Package for Social Scientists (SPSS) soft ware version 16. The data were stratified according to women age at quinacrine sterilization and duration of quinacrine sterilization to control confounders. All variables in the study were expressed as frequencies and percentages. The continuous variables like age were reported as mean, standard deviation and standard error. The risk estimation was performed by constructing 2×2 tables, calculating crude odds ratio and 95% confidence intervals. P values were described to show significance. The p value less than 0.05 was considered significant. The missing data were excluded because it was less than 5% for all variables. The chi-square-test was used to test for differences in the distribution of quinacrine sterilization failure as well as endometrial growth stratified by age and duration of quinacrine sterilization.

The multivariate analysis was performed to calculate adjusted odds ratio. The confounders identified were age, parity, obesity, socioeconomic status, and general heath status. Age was coded as more than 35 years, equal or less than 35 years, parity was coded as less than 5 children, more than 5 children. Obesity was coded as Body mass index more than 30, less than 30. Socioeconomic status was derived from social status score developed by the National Institute for Social Research based on income, employment, and level of education. These scores were divided into low socioeconomic status (below 25th centile), medium (between 25th and 75th centile), and high socioeconomic status (above 75th centile). The general health status was coded as good if no chronic illness was present and worse if chronic illness (disease more than 3 months) present.

## RESULTS

The random sample of 540 quinacrine sterilized females was selected. The calculation of statistics showed the mean age at quinacrine sterilization was 38.5 years, standard deviation 6.517, standard error 0.461. The sterilization failure rate 10.74% was present in the study.

***Demographic features of study participants: ***The demographic features showed that the most of the females were more than 35 years old when sterilized with quinacrine (80%), living in rural areas (67%), poor (51.85%), unemployed (89.7%) and uneducated (65%). (Table-I)

***Endometrium on transvaginal ultrasonography: ***The transvaginal ultrasound showed endometrial adhesions in 15% and endometrial thickness less than 5mm in 13% females after receiving quinacrine sterilization.

The endometrial patterns found in present study were regular and smooth endometrium with homogenous images in 86% (n= 466) women, irregular endometrium with heterogeneous images on transvaginal ultrasound in 9.4% (n =51) and endometrial growth with high level echoes in 4.2% women (n= 23) after quinacrine sterilization. (Table-II) 

The histological findings included adenocarcinoma in two and hyperplasia in two women. One woman had simple hyperplasia and other had complex hyperplasia. In simple hyperplasia, there was increased number of proliferative glands. The glands were large in number, crowded and separated by stroma. In complex hyperplasia with atypia the glands were present ‘back to back’. There was marked cytological atypia, pleomorphism, hyperchromatism and abnormal chromatin patterns. The adenocarcinoma was seen with irregular glands lined by malignant columnar epithelial cells.

***Univariate Analysis:*** The univariate analysis had shown that the age equal or less than 35 yrs at quinacrine sterilization was 9.63 times more at risk of sterilization failure than the age more than 35 yrs at quinacrine sterilization(OR 9.63, 95% CI 5.15-18.09, p<0.05). It was found statistically significant. (Table-III)

The quinacrine sterilization duration more than ten yrs was 7.59 times more at risk of endometrial growth than duration of quinacrine sterilization less or equal to ten years. (OR 7.59, 95% CI 2.98-19.38, p<0.05). It was found statistically significant. (Table-IV)

***Multivariate Analysis:*** The logistic regression analysis was employed to control the confounders. The multivariate analysis had shown that the 35 years or younger age when received quinacrine sterilization was 8.08 times more at risk of sterilization failure than the age more than 35 yrs. (OR 8.08, 95% CI 5.01-10.63, p<0.05). 

The quinacrine sterilization duration more than ten years was 9.39 times more at risk of endometrial growth than duration of quinacrine sterilization of ten years or less. (OR 9.39, 95% CI 5.94-17.26, p<0.05). It was found statistically significant. (Table-IV)

## DISCUSSION

The quest for a secure and effective contraceptive has puzzled the pubic for centuries. Quinacrine sterilization was hypothesized to cause mutagenesis and carcinogenesis in women after intrauterine insertion. Theoretically, quinacrine was described to bind with deoxyribonucleic acid (DNA). Therefore the risk of genetic mutations and carcinogenesis was increased.^13^ Contrary to this, the latest studies had documented the anti cancerous role of quinacrine by reversing multidrug resistance in cancer cells.^14^ The expert panel of WHO had recommended surveillance regarding adverse maternal outcomes and endometrial abnormalities in women after quinacrine sterilization.

**Table-I:** Demographic features of female quinacrine sterilization

***Variable***	***frequency***	***percentage***
***Age at quinacrine sterilization***		
35 years or less	106	19.6%
More than 35yrs	434	80.3%
***Residence***		
Rural	362	67%
Urban	178	33%
***Employment***		
Employed	59	10.93%
Unemployed	481	89.07%
***Poverty***		
Yes	280	51.85%
No	260	48.15%
***Literacy***		
Literate	189	35.1%
Illiterate	351	64.9%

**Table-II:** Frequency distribution of study variables

***Variable***	***Frequency***	***Percentage***
***Duration***		
1. 10 yrs or less	463	85.74%
2. more than 10 yrs	77	14.25%
***Sterilization failure***		
Yes	58	10.74%
No	482	89.26%
***Endometrium***		
1. regular	466	86.29%
2. Irregular	51	9.4 %
3. growth	23	4.2%
4. Endometrial carcinoma	2	0.37%
5. Endometrial hyperplasia	2	0.37%

**Table-III:** Quinacrine sterilization age and sterilization failure

***Age***	***Sterilization failure present***	***Sterilization failure*** ***absent***	***Odds ratio.*** ***(95%CI)***	***Adjusted Odds ratio.*** ***(95%CI)***	***Chisquare***	***p-value***
35 yrs or less	36	70	9.63 (5.15-18.09)	8.08 (5.01-10.63)	74.18	0.00
More than 35yr	22	412				

**Table-IV:** Quinacrine sterilization duration and endometrial growth

***Duration***	***Endometrial growth*** ***present***	***Endometrial growth*** ***absent***	***Odds ratio*** ***(95%CI)***	***Adjusted Odds ratio (95%CI)***	***Chi-square***	***P value***
More than 10 yrs	12	65	7.59 (2.98-19.38)	9.39 (5.94-17.26)	28.25	0.00
10 yrs or less	11	452				

In the present study mean age at quinacrine sterilization was 38.5 years, standard deviation 6.517, standard error 0.461. Suhadia and Sokal had described follow up findings of quinacrine sterilization in females having mean age 33.2 years, standard deviation 9.75 and mean age 36.4 years, standard deviation 6.61 respectively.^15^ The risk of sterilization failure was found more in 35 years or younger age in recent study.

The sterilization failure rate in quinacrine sterilization was found 10.74% in Faisalabad. In Vietnam after 5 years follow up of different sterilization methods, quinacrine sterilization had the highest failure rate 13.3%, tubectomy had the lowest failure rate 1.0% and followed by 4.1% with vasectomy.^16^^,^^17^ In Vietnam, the double insertions of quinacrine pellets were used to sterilize women while in Faisalabad single insertion of quinacrine pellets was the preferred method. The training of health care workers had shown to decrease sterilization failure rates from 5.4%(SE2.3) to 0.9%(SE0.4).^6^

The endometrial patterns found in present study were regular and smooth endometrium with homogenous images in most of the women, irregular endometrium with heterogeneous images on transvaginal ultrasound in 10% and endometrial growth with high level echoes in 4.2% women after quinacrine sterilization. Ferriera had described the endometrial patterns on transvaginal ultrasound after quinacrine sterilization as irregular high-level echoes, or variable echogenicities showing irregular endometrium. The irregular endometrial lining was produced by intrauterine adhesions in 6.4%. The decrease in endometrial thickness as measured from one echogenic border to another border across the cavity of the uterus was observed in 11% women.^12^

The insufficient family planning is the leading cause of maternal mortality in developing countries.^18^ In this study there was endometrial hyperplasia in two (0.37%) and endometrial carcinoma in 2(0.37%) after quinacrine sterilization. In an international study the women were investigated for endometrial abnormalities through transvaginal ultrasound and subsequent histopathology for confirmation and it was found that the chances of histopathological evidence of endometrial abnormalities were increased if history of irregular bleeding was present along with abnormal endometrium on ultrasound.^19^ In present study the biopsy was performed in women after quinacrine sterilization that had irregular, non homogenous, suspicious images on transvaginal ultrasound and suffering from irregular uterine bleeding to increase the yield on histopathology. 

Another research showed the difference in thickness of endometrium in cancer and hyperplasia were found statistically significant.^20^ In a study it was found that measurement of endometrial thickness was important in diagnosing abnormal endometrium with irregular uterine bleeding.^21^ Similarly in the present study the cut off value of 5mm for endometrial thickness was used.

In international studies conflicting evidence was presented about endometrial tumors and growths after quinacrine sterilization. Many experimental studies were conducted on animals to provide evidence in this regard. In present study the quinacrine sterilization duration more than ten years was 7.59 times more at risk of endometrial growth than duration of quinacrine sterilization less or equal to ten years. However, research studies in Chile showed no significant evidence of increased risk of reproductive tract cancers for this method.^22^

Sokal and his colleagues, in a follow-up study of quinacrine female sterilization in Vietnam, had shown no association between carcinogenesis and quinacrine sterilization. They also demonstrated that the number of hysterectomies for endometrial abnormalities was much higher (8%) in females surgically sterilized than in quinacrine sterilization i.e.0.5%.^23^ Hypertension and adverse maternal outcomes were found after quinacrine sterilization.^24^^,^^25^

The strength of present study was that the population based study was conducted and endometrial abnormalities of quinacrine sterilization were investigated by transvaginal ultrasonography and histopathology. Further research studies should be designed for better policy making regarding sterilization and reproductive health.

## CONCLUSIONS

Most of the women had normal, smooth, regular and homogenous endometrium after 6-17 years of quinacrine sterilization. The cases of irregular endometrium, endometrial adhesions, growths and sterilization failure were also found after quinacrine sterilization. The sterilization failure rates were more in women younger than 35 years during quinacrine sterilization. The risk of endometrial growth was more after 10 years duration of quinacrine sterilization. Thus, quinacrine sterilized women should be followed for long term side effects.

## Contribution of authors:

SA conceived, designed the research, did data collection, statistical analysis, writing and editing of manuscript.

MHB designed, did histopathology, research writing, interpretation of results, critical analysis, review, and final approval of manuscript.

SA takes the responsibility and is accountable for all aspects of the work in ensuring that questions related to the accuracy or integrity of any part of the work are appropriately investigated and resolved.

## Notes

***Source of funding:*** No research grant was received for this study and there is no conflict of interest.
